# Spirit of the British and American Periodicals

**Published:** 1839-04-01

**Authors:** 


### Spirit of the British and American Periodicals.

### Virtues of the Iodide of Potassium.

Mr. Laycock, in a communication to our contemporary, the Medical Gazette, sets forth in strong colours the use, if not the utility of the iodide of potassium.

The quantity of iodide of potassium consumed in the hospital for the last three years, is as follows :—In 1836, 12½ oz. in 1837, 63½ oz. ; in 1838, 75 oz.

It would seem that Mr. Russell the junior surgeon prescribes sarsaparilla and *not* iodide of potassium, *because* he is unable to understand the rationale of the latter—while Mr. Champney prescribes the iodide in similar cases, because, we presume, he is unable to understand the rationale of the former. This reminds us very much of the two sects in Babylon, one of which made it a cardinal point to enter the temple with the right foot foremost, while the other denounced damnation on all who did not advance the left foot.

Mr. Laycock relates four cases. We shall only quote the fourth.

*Case* 4.—"This is another 'sarsaparilla' case. Mary Brown, thin, bloodless, and with nasal voice, has ulcers in the throat, and nocturnal pains. She has taken mercury for a syphilitic disease. She was admitted an out-patient on August 20th, 1838, and took first the simple decoction of zarsa ; then Hudson's syrup, with lime-water ; and lastly, the decoction, with half an ounce of the extract to each pint. She got no better, and was made an in-patient on Nov. 1st. For two or three weeks she took five grains of the iodide of potassium in a wine-glassful of the decoction of zarsa, and in two or three days there was a manifest improvement; the appetite returned, the nights were good, and the ulcers in the throat looked clean. She was convalescent on December 22d, when she was made an out-patient, and in a few days was discharged cured. She has had no relapse up to the present date."

Upon this and on the preceding cases, Mr. Laycock makes some observations, which, perhaps, require notice. He says :—

"The iodide of potassium is a specific in such cases as 1 and 2, if it be genuine, and given regularly and in sufficient doses. The shortest time in which I have seen an ulcerated throat healed is 18 days. In cases in which there is sallowness, emaciation, syphilitic ulcers, and where mercury has previously been given, I have never seen it fail. When the osseous tissues are affected, the health is as rapidly restored ; but, of course, the dead or careous bone requires the usual time for exfoliation.

In venereal eruptions it has not been found quite so successful as in the preceding cases, but required its action to be quickened by small-doses of blue-pill or calomel.

The iodide has been very freely administered in other forms of disease. In cutaneous eruptions it has been signally successful. A youth, aged 19, the son of a country shoemaker, came into the hospital with a chronic impetiginous eruption of sixteen years' standing. Every kind of medicine usually prescribed had been tried without success, in addition to a course of the Harrogate waters. He was admitted under the care of Mr. Champney, on Feb. 16, 1837, and was dismissed cured April 16. He took three grains only of the iodide, three times a day, for the first month, and his cure was proportionally slow. He took blue-pill and extract of colocynth occasionally, and used an ointment composed of half a drachm of the chloride of mercury, and an ounce of the dilute ointment of the nitrate of mercury.

Given in ptyalism it seemed to have the effect of increasing the discharge; but it has never excited the salivary glands when given alone. Dr. Simpson has prescribed it with considerable success in hypertrophy and other diseases of the heart, which constitute a large proportion of our cases. It has also been given with success in rheumatic, neuralgic, and paralytic affections. It has been tried with varying success in visceral enlargements, and almost every form of chronic organic disease."

He adds:—

"The superiority of iodide of potassium to zarsa, in the treatment of secondary syphilis, is now placed beyond all doubt by so much concurrent testimony; and it would be a worthy object of enquiry to determine how far it may be made to supersede the latter expensive medicine in common cases in which it is now freely used, as in diseased joints and scrofulous abscesses. The period of stay of venereal cases in hospital is shortened, on an average, at least two-thirds, when treated with the iodide."

We would suggest to Mr. Laycock, that a little less confidence in his own opinions, and a little more respect for the opinions of others, would not be unbecoming. Mr. L. dogmatises pretty boldly, and yet some exception may be taken to his statements.

We do not hesitate to say that he exaggerates the powers of the iodide of potassium. It is very far from a specific in such cases as the one that we have quoted. No doubt it is a valuable medicine, no doubt it often relieves, sometimes cures almost magically, but no doubt it not unfrequently fails. We have at this moment several cases of this kind under our care which have been only temporarily, or not at all benefitted by the iodide.

Mr. Laycock says that it is not *quite* so successful in the *venereal* eruptions! What are those eruptions? In the majority of the cases of genuine venereal eruption we venture to affirm that the iodide is not successful *at all.*

In another place Mr. Laycock remarks that the superiority of the iodide over sarsaparilla in the treatment of secondary syphilis is placed beyond all doubt. The truth is, that neither one nor the other goes far towards curing secondary *syphilis.* That, after all, requires mercury. Sarsaparilla is an excellent adjunct to the latter, because it maintains the health, not because it exerts much influence over the disease. In some instances, the iodide is a good adjuvant too, but not so good, in our opinion, as the zarsa.

It is in the cachectic cases, those cases which either result from the abuse of mercury, or from the compound agency of syphilis and mercury on a peculiar habit—it is in those cases, we say, that both the iodide and sarsaparilla are peculiarly serviceable. It is difficult to say which is most so: an injury would be inflicted on medicine were it deprived of either. With some persons the iodide acts best—with others sarsaparilla—with most, it is better to combine the two.

We fancy that we perceive, what usually happens, a very indiscriminate and therefore a mischievous use of the iodide. It is astonishing what a disposition men's minds have to run only in a groove. Some people prescribe nothing but creosote one moment—nothing but iodide the next.

---

### State of the Faculty and of Physic in Algiers.

Physic would seem to be at a low ebb in Algiers. The doctors certainly are so. The following extract from an extract in the Medical Gazette will exhibit the state of things in that country.

"Dr. Bohn first introduced vaccination, and practised it in the family of the deposed Dey himself, who, however, did not give him a princely fee; and, generally speaking, people are here unwilling to give aught to physician or apothecary.

As to the native physicians, the Dey had a kind of protomedicus, who decided medico-legal questions, and created other physicians for a few piastres, without being exactly able to read and write. If a man was able to shave well, if he could compound a plaster, and cure a hurt, he bought the privilege, and prescribed at his own pleasure the whole contents of any of the six Moorish apothecaries' shops; bark with or without theriaca at all times; and in all fevers, opium, sarsaparilla, calomel, pimento, cantharides, and opodeldoch. Ismael Ben Mehmed enjoyed the greatest share of public confidence; he gave Dr. Schönberg an extract from the Arabic work of Ben Huesina, who lived 700 years ago, and a catalogue of his own drugs. His shop, the largest in the town, contained 70 jars, 30 bottles, 20 boxes, and several drawers. He obtained medicines from abroad, prepared others himself, and possesses a still and retort. He is afraid of mercury against syphilis, and thinks he can do without it.

Ismael Ben Mehmed is acquainted with remittent and intermittent fevers, and their varieties. His surgical apparatus consisted of a common case of dressing instruments."

---

### Dr. Corrigan's new Mode of effecting the Inhalation of Iodine, &c. in Pulmonary Diseases.

Dr. Corrigan patronizes the inhalation of medicated vapour in pulmonary diseases, but justly objects to the inhalers previously used. He offers the following suggestions.

That inhalation as a remedial process, may obtain a fair trial, it is requisite,

1st. That the apparatus should be simple in its construction, and easily kept in order.

2nd. That it should be capable of keeping up a supply of vapour for any length of time, and that the evolution of the vapour should be steady, and should be easily regulated.

3rd. That it should also furnish a sufficient supply of aqueous vapour, to prevent any irritation of the larynx or lining membrane of the air tubes.

4th. And most important of all, that its employment should entail neither trouble nor fatigue on the invalid.

The apparatus he patronises is thus described :—

There is a light open iron wire frame, about eighteen inches in height; at the bottom is a spirit lamp, At the proper height above it, is an evaporating porcelain dish, about six inches diameter. Above this is a glass globe with its neck downwards. In the neck of the globe is a cork bored, and through the opening is drawn, moderately tight, a short plug of cotton wick, such as is used in a spirit-lamp; in the glass globe opposite the neck is drilled a pin hole, to allow air to pass in, according as the fluid within drops out, through the neck. To use it, the porcelain dish is filled with hot water, the spirit lamp is lighted, and as soon as the water in the dish has begun to boil, the glass globe containing the tincture of iodine (if this be the substance used) is placed as shown in the sketch. The rate at which the fluid in the globe shall percolate the cotton wick, and drop into the hot water underneath, is easily regulated. If it do not drop with sufficient rapidity, one or two of the threads of the cotton are to be removed. If it drop too rapidly, this is corrected by pressing in the cork more tightly, or introducing one or two additional threads of wick.

This apparatus fulfils, I think, all the conditions required. It is simple in construction and most easily regulated; there can be no sudden and injurious evolutions of vapour from it but drop by drop, the evolution gradually, and steadily goes on; and the air which the patient is breathing, may be maintained in any required degree of impregnation, while the impregnation can be kept up for any length of time. The medicated substance employed, is always vaporized with a sufficient quantity of aqueous vapour, to prevent any irritation of the larynx, or lining membrane of the air tubes; and lastly, employment of the apparatus for any duration, entails neither trouble nor fatigue on the invalid.

### UREA IN THE BLOOD IN CHOLERA.*

In a case which presented all the features of malignant cholera, and which proved fatal on the 12th day, the deficiency of urea in the blood induced Dr. Rainy of Glasgow, to investigate the state of the blood.

Four ounce measures of blood were taken from the larger vessels and heart; it was partly fluid, partly in small coagula. As the serum could not be separated in sufficient quantity for examination, the whole was mixed with 12 ounce measures of alcohol, well stirred for some minutes, and then allowed to digest for a day at a moderate temperature. The albuminous and colouring matter was precipitated in reddish brown flakes. The alcoholic liquor partly floated on the top. The mixture being thrown on a filter of fine cotton cloth, ten ounce measures of fluid passed through, by the aid of gentle compression, the rest being retained by the spongy precipitate. This filtered fluid was transparent, and almost colourless. It was evaporated at a temperature not exceeding 160° Fah. During this process it became turbid, and deposited a considerable quantity of fluid oily matter, which was separated. When reduced to the consistence of a thin syrup it had a decidedly urinous smell; and a minute portion being tested, yielded distinct pearly scales with nitric acid and with oxalic acid. The syrupy fluid was still turbid, apparently from the presence of oily matter, probably phosphoric fat. In order to separate this matter the extract was diluted with a little water, to render it perfectly fluid. It was then agitated with a small portion of æther, which dissolved the oily matter, and left the watery fluid colourless and almost transparent. This fluid was again evaporated, at a very gentle heat, to the consistence of a thin syrup; and nitric acid being added, there was a slight effervescence, followed by a deposition of pearly crystalline scales. These, being compressed between folds of filtering-paper and dried, weighed $5\frac{1}{10}$ grains. They had all the characters of the nitrate of urea, and, according to Prout's analysis, may be considered equivalent to $2\frac{7}{10}$ grains of urea.

This quantity, then, was actually separated from four ounce measures of blood; but as the whole mixture of blood and alcohol measured 16 ounces, and the filtered liquor measured only 10 ounces, it is evident that the filtered liquor contained only $\frac{10}{16}$ of the urea present in the blood. From these data it will follow that the whole urea actually present in the blood amounted to $2\frac{7}{10} \times \frac{16}{10} = 4\frac{3}{10}$ grains, or rather more than one grain to each ounce measure of blood, without making any allowance for the small quantity remaining in the fluid, to which the nitric acid was added.

---

### MR. SMEE ON MOULDING TABLETS FOR FRACTURES.†

Mr. Smee has been making some experiments with the view of ascertaining the best method of forming moulding tablets.

Having frequently noticed that the composition of gum arabic and whiting, when dry, possessed great hardness and toughness, and yet was so free from brittleness that it could scarcely be pounded in a mortar, he was determined to ascertain how far it would answer to make tablets, which might be used for extemporaneous splints.

For this purpose a piece of coarse sheeting was copiously brushed over on one surface with a thick solution of gum, after which it was covered with a composition made by rubbing whiting with mucilage, continually adding the powder

---

* Med. Gaz. Jan. 5, 1839. † Med. Gaz. Feb. 23.

until the whole was of the consistence of a thick paste : a second piece of sheeting was now rubbed over on one side with the solution of gum, and the moistened side applied upon the composition with which the piece of sheeting had been covered, and we thus had two thicknesses of sheeting with an intervening layer of the composition of mucilage and whiting, the thickness of which may be increased or diminished as strength or lightness is desired. The whole was then dried, and formed a tablet about the thickness of slight pasteboard.

"This experiment succeeded beyond my most sanguine expectations; for, whilst the tablet remained dry, it was exceedingly hard, and, when sponged over with a little warm water, became so yielding, that, by moulding it with the fingers, a cast could be taken of any part of the body. The hand and knuckles were defined with great accuracy, and I succeeded, by a little management, in taking a cast of the greater part of the face. It is sometimes advisable not to allow the substance to dry upon the part on which it is moulded; but after the depressions and elevations have been traced with the fingers, it should be carefully removed, and partially dried before the fire ; and as soon as the texture is sufficiently dry to retain its shape, it may be placed near a stone, or even on the hob of a grate, without fear of corrugating or becoming otherwise deformed. In most cases, however, this drying is quite unnecessary, it being requisite only to envelop the moist tablet with a bandage. A cast thus taken is extremely hard and tenacious, so that when not much thicker than a wafer, it may be struck violently and repeatedly against any hard substance, and not be destroyed. It possessed but slight flexibility, and, after having been bent, it returned to its previous form, shewing considerable elasticity. It was neither liable to be torn nor broken ; and lastly, it possessed the advantage of lightness combined with durability."

The solution of gum which was found most adapted contained 10 or 12 oz. of gum to the pint of water. Coarse old linen sheeting forms the best kind of cloth.

The application of these tablets is rather extensive; they may be used with great advantage for all fractures of the metacarpal bones; also for those of the fore-arm, or even for the humerus.

When the humerus is fractured, the method which has been adopted is to cut a piece of paper somewhat into the shape of the required splint. It should cover a portion of the pectoralis major, and extend as high as the bend of the neck, and include the whole of the scapula. From this broad plate a piece descends to the bend of the elbow, and should be sufficiently wide to cover about two-thirds of the outer part of the arm. The paper is then placed on one of the prepared tablets, which is cut to a similar shape. The piece thus prepared is moistened until it becomes perfectly soft, and it is then moulded on the arm and neck. From the general shape of these parts, there will be found a superfluity of substance about the deltoid which must be pinched up and turned down, so as to form a fold over the other part. The splint then may be in a degree dried, and its inner surface lined with lint. The whole is to be enveloped with a starched roller.

The mode in which I have made splints for the leg, is first to obtain the exact shape by drawing a piece of sheeting or paper round the leg, and marking the part which corresponds to the tibia for the whole length of the leg, and continuing the line on the foot to the extent that it may be considered necessary to cover.* By this means, it is apparent that the exact size of the limb is obtained ; but as the leg is to be enclosed by two splints, it becomes necessary to divide the

* "Either splint should overlap the heel and under surface of the foot in cases where they are used immediately after the accident, but where their application is delayed, this is of no importance."

cloth into two, which will give an exact pattern of either splint. These splints are to be moistened and moulded, and after being first lined with lint, or leather, the whole is to be enveloped by a roller soaked in boiled starch."

Of course those splints are not to be applied until swelling and inflammation have subsided. Mr. Smee's composition deserves a trial.

---

OBSERVATIONS ON THE EFFICACY OF THE SALTS OF SILVER IN THE TREATMENT OF SECONDARY SYPHILIS. BY WILLIAM P. JOHNSTON, M.D. OF GEORGIA, AND JAMES TRUDEAU, M.D. OF LOUISIANA.

These physicians communicate to our American contemporary, the Medical Examiner, the results of experiments by M. Biett, on the use of the salts of silver in primary and secondary syphilis.

These salts were recommended by M. Serre, of Montpelier, in 1813. M. Serre published twenty-five cases, of which we will only say that they by no means proved the points he brought them forward to establish. The Reporters, in the paper before us, relate five cases from the practice of M. Biett. We shall not specify them, but content ourselves with the following brief summary of M. Biett's conclusions—conclusions, as regards the salts of silver, which coincide exactly with our own experience on the subject.

"In the above cases, which we have been obliged to condense very much, it will be seen, that the salts of silver have invariably failed in producing the slightest modification in the disease. Mr. B. in his Clinical Lectures, says that he has employed the different preparations of silver recommended by M. Serre, in a vast number of cases, and that he has continued the treatment until the amount taken was thirty grains, without ever having seen it produce 'beneficial effects, and in some cases to the manifest injury of the patients.'

In conclusion we beg to say a few words of the remedies which, in the hands of Mr. B. have been so successful in the treatment of this form of disease. The proto-iodide of mercury, introduced by him in the treatment of syphilides, is that to which he gives the preference. It is given in the form of a pill in doses of one grain, with an equal quantity of lactucarium, night and morning. This remedy, in his hands, rarely produces salivation. He employs, in conjunction, the vapour baths, which he regards as a powerful agent in completing the cure and restoring the healthy functions of the skin. Opium plays an important part in the formulary of Mr. B. He employs it in cases where irritation of the stomach and bowels has been produced by the metallic preparation, or where the system generally is in a state of nervous irritability. His usual practice is to give one grain of the watery extract, morning and evening, during a month, and then he resumes the treatment with the proto-iodide. Mr. B. says he has often seen opium produce a decided amelioration in the disease, when all other remedies have failed. He has also remarked cases where a modification produced by the proto-iodide has continued under its use until a complete cure was effected. The liquor of Van Sweiten and sudorific drinks, are sometimes employed. Simple cerate is the only local application for the ulcers, and even this is but rarely used. In support of the treatment of Mr. B., which, from having long and carefully observed it, we can recommend, with the greatest confidence, we will quote the authority of M. Ricord. He says, 'the preparation to which I now give the preference, not only in the treatment of the secondary, but also in that of the primitive symptoms, is the proto-iodide of mercury.' Opium, he says, alone, or more frequently combined with other remedies, is one of the most useful agents in the treatment of this disease, and ought never to be neglected."

---

### Observations on Various Diseases. By Robert James Graves, M.D.*

These observations, like all from Dr. Graves's pen, are possessed of interest and value. We shall select some.

1. *Neuralgia of the Larynx.*—This occurred in a young lady originally of vigorous constitution, but latterly suffering from menstrual irregularity and hysteria. The laryngeal affection had been considered to be inflammatory in the country, and had been treated with purgatives, leeches, blisters, antimonials, and finally mercurialization. No relief had been obtained, and she came to Dublin where she was placed under the care of Drs. Marsh and Graves, and Mr. Barker. The pain had become almost constant when we first saw her, but was by no means violent, except now and then when it used to become suddenly aggravated. These paroxysms of pain could not, even properly speaking, be called violent; they were however, distressing, amounted to a most annoying feeling of distress about the whole region of the larynx. There was no external tenderness, and the internal fauces were healthy. These gentlemen considered it an hysterical nervous affection. It was chiefly remarkable for a change of tone and weakness in the voice which invariably attended the paroxysms, shewing that the *rima glottidis* and the *chordæ vocales* were the parts chiefly implicated.

They first gave doses of carbonate of iron, which had the effect of rendering the attacks periodic. Every morning, at *ten o'clock to the minute,* the paroxysm commenced. The dose of iron was now increased, afterwards sulphate of quinine, and finally arsenic were employed, but without any corresponding improvement. The degree of suffering became, indeed, less severe, and its duration less protracted, but it appeared extremely doubtful whether the improvement was not owing more to time than to medicine.

" Under these circumstances we thought it prudent to desist from all active treatment, and we recommended change of air, scenery, and the use of chalybeate mineral waters. I believe she still continues to suffer, but in a less degree. A year has now elapsed since I last saw her. This case affords a striking example of the curious fact, that medicines administered for the purpose of relieving a disease more or less fluctuating or remittent in its character, will sometimes render it strictly periodic, with marked paroxysms and free intervals. Having produced so striking an effect with our remedies, we are apt to calculate with confidence on still further improvement, and we increase the doses of tonics with boldness and full of hope; disappointment, however, here awaits us, for no tonic will be found capable of effecting any further alteration or shortening of the fit. In such cases we cannot be too much on our guard, lest we injure the constitution by too frequent attempts to procure a diminution of suffering."

We have often thought that what appear to be the effects of medicines in these cases, are often not really due to them as particular drugs. The morale of the patient is disturbed, and it is difficult to separate mental affections from physical disturbances. It is a thousand to one that, in another case, the carbonate of iron would not give rise to periodicity of attack. We doubt whether the carbonate of iron, *as* carbonate of iron, did so in the case before us. Practitioners should beware how they draw conclusions on the effects of medicines in cases (more particularly in insulated cases) of hysteria.

2. *Neuralgia of the Testicle.*—Dr. G. has seen two examples of this painful

---

* Dublin Journ. Jan. 1839.

affection within the last year. The first was a young gentleman of highly irritable nerves, who had studied hard and dissipated much ; in him the paroxysms of pain did not observe any very marked period, but returned daily at uncertain intervals, which grew shorter and shorter, until at last he had scarcely any respite day or night. There was no fever, and not the slightest appearance of local congestion or inflammation. When attacked with a paroxysm the patient would throw himself on the floor, and roll about in the greatest agony, covered with a cold perspiration. This case yielded to large doses of carbonate of iron freshly prepared, and frequent inunction of the testicle and cord with belladonna ointment. The second case of neuralgia of the testicle occurred in a gentleman who laboured under neuralgic pains, decidedly of a gouty nature. In him the pain of the cord and testicles used to come on every afternoon about four o'clock, and continued for several hours. The pain, though considerable, did not approach the degree of agony experienced in the first case. It was at times, however, so severe as to compel him to groan aloud. This neuralgia of the testicle disappeared after a few days, and was replaced by a violent gouty pain in the loins and right hypochondrium. The latter yielded to the usual local treatment and the use of colchicum internally.

We would observe that neuralgia of the testicle, or " irritable testicle," occurs under various circumstances. Perhaps one of the most common causes is abstinence from sexual intercourse, after previous indulgence in it. A short time ago, we were in attendance on a gentleman, of a sanguineous habit and of rather strong passions, who became a widower. The consequence was irritable testis under which he laboured at intervals for eighteen months. He then married, and has never since had any pain in the testicle. The pain in this gentleman was sometimes exceedingly severe, and, what is curious, he generally experienced relief from the passage of a large instrument into the bladder. Yet he had not stricture, though he usually experienced some irritability of bladder, at the time when his testicle plagued him.

In persons who are recovering from gonorrhœa, and who have merely slight discharge, or gleet, a certain degree of irritability of the testicle is not unfrequent. We have at this time under our care a young gentleman, who suffers in this way in both testes. It is difficult, in these cases, to determine, whether it is the condition of the urethra, or the abstinence from sexual intercourse, or both, which gives birth to the affection.

We have seen in gouty individuals, a degree of tenderness, almost amounting to positive neuralgia in the testis. But we never saw it precede a distinct gouty attack. Dr. Graves's is a well marked instance of this.

But irritability of the testis, and even positive inflammation, are well known to wait on affections of the kidney. And this organ is so frequently disturbed in gout, or in the gouty, that we cannot wonder at the latter being prone to testicular disorder. And it must be recollected that the gouty are usually plethoric, whilst, plethora, so far as we have seen, disposes to irritable testis, more, perhaps, than any other condition.

It is absurd to suppose that a complaint depending on causes of different descriptions will universally yield to one remedy. We shall not touch on the methodus medendi, further than by remarking that there is one local remedy which is almost always useful—belladonna,—whatever other measures may be requisite.

3. *Gout and Sinapisms.*—" Within a short period of time I have seen three remarkable examples of the relief which vital organs may experience when gout appears in the extremities. A publican applied to me with violent pain in his stomach, which came on every evening, and lasted many hours in spite of every remedy. In a day or two he got a violent attack of podagra, and had no more internal suffering. A gentleman whom I attended with Mr. Barker, was attacked with cerebral symptoms and indistinctness of vision and utterance. We feared hemiplegia; the next day he got severe podagra, and was able to speak perfectly well, and see distinctly. He was 75 years of age. At the very same time I was attending, with Mr. Colles and Mr. Haffield, a robust and powerfully-made gentleman, aged 74, who having had symptoms of flying gout, and shortly after a bowel-complaint, made use of the salt water plunge bath. This imprudent act brought on a violent and nearly fatal hemoptysis. He was bled twice, and got the usual styptics with relief, but his improvement became much more rapid when gout appeared in both his feet."

Dr. Graves thinks that sinapisms which act slowly are the best. To fix gout in a part, says he, e. g. in the foot, our application must act much more gradually, and must excite the deeper-seated tissues. These objects may be obtained by mixing one part of strong and fresh ground mustard powder with three of flour, and adding as much treacle as will convert them into a viscid paste, which may be spread like a plaster on linen, and applied to the part. This will be borne for four or six hours, and will cause a redness which will last a whole day. The proportion of flour may vary according to circumstances.

4. *Mr. Cusack's Mode of Applying the Nitrate of Silver to Enlarged Amygdalæ.* The solid stick of lunar caustic, or some of the latter in powder, and placed in a proper instrument, must be kept steadily pressed against a particular spot of the enlarged gland; two three, or five seconds will suffice to secure the formation of a small eschar, which falling out, will leave in the part, when healed, a slight depression like the largest pit formed by a small-pox pustule. When this has been effected, which is usually in about five days, a similar proceeding must take place with the other amygdala; and so on with each, turn about, until the desired reduction of size has been accomplished. When the glands are large, this process usually requires about six months; it is slow but sure; and must be intermitted when any accident gives rise to temporary sore throat or to catarrh.

---

### Dr. Law on the Exhibition of Mercury in Minute Doses.

Before we make any observations, we shall quote the following case—and remarks.

"A young man recently came under my care affected with syphilitic rheumatism. His pains were very distressing, and completely interfered with his sleep. He had undergone a variety of treatment, but without any relief. Just before he became my patient, he had taken fifteen grains of blue pill daily for six weeks, without his mouth becoming sore, or his pains experiencing any benefit. I found that while he was under this mercurial treatment, he was neither restricted in diet, nor confined to the house; I determined to try mercury with him, but under different circumstances. He was put on low diet; and in order to insure his remaining in bed, his clothes were taken from him; he was then directed to take two grains of calomel, and a quarter of a grain of opium, thrice daily. He had only taken twelve grains of calomel, when his mouth became sore, and salivation ensued. His pains then began to yield, and soon disappeared altogether. Thus, with twelve grains of calomel, exhibited with attention to circumstances calculated to promote the action of the medicine, we succeeded in accomplishing what fifteen grains of blue pill, exhibited daily for six weeks, (630 grains) but without such attention, failed to effect. We were quite sure when he came under our care that he was not under the influence of the mercury he had previously taken. The result of this case, with many other similar ones, confirmed us in an impression that we had long entertained, that there is not in the *Materia Medica* an agent whose just pretensions are more compromised by a slovenliness and want of care in its exhibition, than mercury. And although, in some cases, the peculiar circumstances under which the medicine is exhibited, prevent the conditions of restricted diet and confinement being complied with; and, therefore, the physician prescribes it under disadvantages, of which, although he is aware, yet he cannot control them; still we believe, from the little importance attached to these conditions when they might be enforced, that most physicians have yet to learn to what extent they modify the action and effects of the medicine. Upon inattention to these circumstances we should charge, in many instances, the complete failure of mercury to affect the system; and in all, that when the system is brought under its influence, so much more of it is required to produce this effect."

Dr. Law is certainly mistaken if he supposes that well-informed physicians and surgeons are ignorant that warmth and low living accelerate and augment the specific action of mercury. There are few amongst us who are not aware of that.

Patients are not usually submitted to this regimen, because as a general plan it would probably rather be injurious than beneficial. Many under such circumstances would become profusely salivated, and we should return to the old regime of spitting-pots and erethismus. The great improvements in the treatment of syphilis have been in discarding confinement and low living, in allowing moderate and judicious exposure to the open air, in enjoining a reasonably good diet, and in combining with the mercury, sarsaparilla or other tonics, which maintain the general health, and certainly do not promote salivation. The benefits of this plan have been displayed on a large scale in the Lock Hospital of London, and we dare say elsewhere, and occasional exceptions do not eat up the rule. That there are such exceptions all men of experience are sensible. Though fully impressed with the value of open air and moderately good living in the treatment of syphilis, yet we now and then meet with instances in which we cannot shut our eyes to the superiority of a contrary mode of proceeding, and in which we enjoin confinement and court salivation.

But Dr. Law proceeds to another inquiry, and has arrived at another result:—*the very small quantity of mercury required to affect the system, when exhibited in minute doses at short intervals.*

" This quantity was much smaller than we could have had any idea of. The first cases in which we made trial of this mode of giving mercury were chronic cases, such as we felt would, without injury or detriment, await the result of our experiment. We made no particular selection of cases, but such as were labouring under affections which we ordinarily treated with mercury. We directed one grain of calomel to be mixed up with a sufficient quantity of extract of gentian to make a mass to be divided into twelve pills, one of which was to be taken every hour. We found, in some cases, salivation produced by twenty-four pills, or two grains of calomel; and seldom were forty-eight pills, or four grains, required to produce this effect. We would say, that thirty-six pills, or three grains, was the average quantity required to effect salivation. We exhibited blue pill in the same way, and found the mouth to become sore from six grains."

Dr. Law specifies many other cases. We will notice two or three.

Anne Carey, aged 46, affected with periostitis of the femur, had her mouth made sore with two grains and a half of calomel.

John Curran, aged 40, labouring under sub-acute rheumatism, with enlargement of the joints of the wrists, was profusely salivated with three grains and a third of calomel: a decided amendment followed.

John Lynch, aged 36, affected with indolent enlargement of both testicles, particularly the right, was ordered a twelfth of a grain of calomel every hour, and to rub ten grains of mercurial ointment on the right testicle every night. He had only taken two grains and two-thirds of a grain, and rubbed twice, when he became salivated. The induration and enlargement of the testicles completely disappeared.

Anne Clare, aged 40, affected with periostitis of the clavicle, was salivated with three grains, and ten-twelfths of a grain of calomel. Salivation continued for a month.

It is unnecessary to swell the list. But the following case may be added to it.

"We anxiously looked out for a case of Iritis to test this method of exhibiting mercury, when one presented itself, in John Gleece, labouring under syphilitic rheumatic pains, with Iritis of the right eye. The conjunctiva was moderately injected, the cornea unusually prominent, and the pupil irregular. We ordered him the twelfth of a grain of calomel every hour. When we paid him our second visit, we found the eye quite clear, no unusual vascularity, and the pupil quite regular. He had only taken eighteen pills, or a grain and a half of calomel; but the gums exhibited no marks of being affected. As the rheumatic pains continued, we determined to persevere in the use of the mercury, and in the same fractional doses, till the mouth became affected. We were surprised to find that this effect was not produced until he had taken one hundred and seventy pills, or fourteen grains of calomel. This seemed to be by much the most refractory case we had met with; however we discovered, that, in order, as he thought, to make assurance sure, instead of complying with our directions of only taking one pill every hour, after the second day, and after experiencing the benefit he received from eighteen, he took forty-eight within twelve hours. So that the case, so far from constituting an exception, by its negative results confirmed our point."

It certainly does require some confidence in hypothesis to believe that the additional quantity prevented salivation. We confess that we are sceptical. Other things being the same we feel a great difficulty in crediting the homœopathic supposition that a quantity minus occasions an effect plus.

But however this may be, the facts stated by Dr. Law are by no means devoid of practical interest. Few, perhaps, supposed that a very small quantity of mercury may by management be made to produce a greater given result than a quantity much larger in different circumstances.

---

### Shortening of the Neck of the Thigh Bone, independently of Fracture, in Early or in Middle Life. By George Gulliver, Assistant Surgeon to the Forces.

In old subjects the neck of the thigh bone is well known to become in many cases shortened, and to form a less considerable angle with the shaft, so that the head of the femur sinks, and the limb is diminished in length. These changes are familiar.

But it is not generally understood that in early life, and at any period antecedent to old age, changes of a similar character may ensue, and a mere contusion of the hip may give rise to interstitial absorption and alteration of the bone, attended with shortening of the limb, and, consequently, simulating fracture. It is to establish the occurrence of such a series of changes that Mr. Gulliver has published the following cases. It must, of course, be most important to be aware of them, for a patient may be much incensed and a surgeon much disgraced, if deformity follows what the latter considered a mere contusion.

Mr. Gulliver relates four cases, but as the two first were not fatal, and afforded no positive evidence of the actual nature of the injury, we pass them over. We shall therefore confine ourselves to the third and the fourth.

*Case* 3. John M'Grath, aged 30, 2d Battalion Rifle Brigade, was admitted into regimental hospital at Malta on the 30th June 1828, with a severe contusion of the right hip, from a fall over a wall twelve feet high, when drunk. No symptoms of fracture presented. He was discharged on the 7th of August following with very slight lameness, but continued to do the active duty required by his regiment, although he occasionally complained of weakness in the injured part. There was manifest protuberance of the right hip, and appearance of shortening of the limb, with an awkwardness in marching. On the 1st August 1830, he committed suicide.

On examination of the body, the neck of the thigh-bone appeared somewhat shortened, and forming nearly a right angle with its shaft, the upper part of the head being just level with the summit of the great trochanter. There was some adventitious bony matter near the trochanter at the basis of the neck, and an increase of density and thickness of the upper part of the shaft. The capsule of the joint appeared uninjured; but the round ligament had apparently been detached from the head of the bone, to which it had acquired a new connexion near to its original site.

*Case* 4. J. Fox, aged 32, after a service of eight years in the West Indies, died of *phthisis,* for which disease he had been two years under treatment in hospital. A long time after his confinement it was observed that his right inferior extremity was emaciated, but there was no note of any affection of the limb previous to his admission into hospital.

At the *post mortem* examination, the right inferior extremity was found, by measurement as in the preceding cases, to be at least an inch and a-half shorter than the other, and the extent between the *pubis* and trochanter of the affected side was diminished in a corresponding manner. The limb was much emaciated, but its position was natural, and the motions of the coxofemoral articulation were not impaired.

Having removed the upper part of the *femur,* Mr. G. found its neck absent. The head was flattened and expanded considerably; it was approximated to the shaft so as to be situated much below the great trochanter. A section of the part was made, when the upper and lower shell of what remained of the neck, was seen to be formed of compact bone, quite equal to the ordinary thickness in this situation, and the reticular texture of the bone was more dense for some distance from the edges, so as to form an indistinct line on either side of the most contracted part towards the centre. The *cancelli* were filled with caseous matter, in some places nearly colourless, in others tinged with dark grumous blood. The acetabulum was diminished in depth, but enlarged laterally, so as to correspond with the altered shape of the head of the thigh-bone. The cartilage of the articulation presented throughout its usual thickness and consistency, and was generally smooth and lubricated with synovia. Mr. G. examined the other thigh-bone, and found its form and condition in every respect natural.

Mr. G. now sought information respecting the history of the case from some of Fox's comrades who had served and come home with him. From them it appeared, that Fox had received a fall about three years before at the island of Nevis, in consequence of which he often complained of pain about the hip, but continued to do his military duty many months after, never having been confined on account of the accident.

The morbid parts described in this and the preceding case, are preserved in the museum of the Army Medical Department, to which the profession have free access through the liberal arrangements of the Director-General.

Mr. Gulliver remarks:—

"The case of Fox appears to afford a well-marked instance of gradual removal of the neck of the thigh-bone in consequence of action induced in that part by injury. From the history of the case it is impossible to suppose that fracture had occurred; and it is improbable that any considerable shortening had taken place previous to his admission into hospital for the thoracic affection, since he performed the duties of a soldier long after the accident, without any lameness apparent to his comrades. We are, therefore, constrained to suppose, that the removal of the neck of the bone had been effected during his very long confinement in hospital for the pectoral disease,—a circumstance not very favourable to the recommendation by Dr. Hawkins of the horizontal posture as a remedy in such cases, and equally adverse to the opinion of those continental pathologists, who attribute this alteration in the neck of the thigh-bone to the gradual operation of the superincumbent weight of the body. But Dr. Knox has long since remarked, that it is unnecessary to have recourse to such an explanation of the cause of interstitial absorption of bone; and in the museum at Chatham this phenomenon is exhibited in the spine of a young man, in which the change took place in the recumbent posture."

We think that these observations and cases should receive attention. Perhaps they are not yet sufficiently precise to give a satisfactory character to the deductions which appear to flow from them. But they go very far towards establishing the occurrence of interstitial absorption of the neck of the thigh-bone, as a consequence of comparatively trivial injuries at any period of life. No unimportant fact. We have already said what we think no more than due to the zeal and intelligence of Mr. Gulliver. We shall be always happy to introduce him to the notice of our readers. It is a grateful task to a man of a liberal mind to extend the hand of encouragement to merit.

---

### Cases of Dissecting Aneurysm.

1. Case of Anomalous Aneurysm of the Aorta, resulting from Effusion of Blood between the Laminæ composing the Middle Coat of that Vessel. By C. W. Pennock, M.D.

2. Account of a Case of Dissecting Aneurysm seen at an Early Stage. By Paul B. Goddard, M.D.

Most of our readers are probably aware of what is meant by "dissecting aneurysm." It is that form of aneurysm, in which the blood becomes effused *between* the tunics of the artery, separating and dissecting them from one another, perhaps for a considerable distance.

A case of this kind lately occurred in the dissecting-rooms of St. George's Hospital, in Kinnerton-street. Unfortunately the parts were too much destroyed before we saw them to admit either of accurate description, or of preservation. But the internal and middle coats of the aorta had given way above the diaphragm, and a large quantity of coagulum extended, immediately beneath the external coat, as low as the bifurcation of the vessel into the common iliacs.

Dissecting aneurysm is sufficiently rare to make well-attested cases of it matters of rational curiosity. And as there are probably some medical men who are unacquainted with its characters, we shall introduce the two following facts from our valued American contemporary.*

*Case* 1. A black woman, aged 75, entered the Philadelphia Hospital, December 20, 1835, with the following symptoms:—

Countenance anxious; position in bed elevated; œdema of the legs and ankles; pulse 90 per minute, full, tense, intermittent; slight muscular movements cause

* The American Journal of the Medical Sciences, November, 1838.

palpitations of the heart; oppression but no pain in the præcordial region. Over the region of the heart percussion is dull in a space, the outline of which corresponds to the form of the pericardium, which extends downwards from the cartilage of the third rib the length of sternum, and laterally, on a line drawn through the nipple, from one inch to the right of the middle line of sternum to the margin of left axilla. Impulse of the heart forcible; rythm nearly natural; first sound roughened, having a rasping sound strongly marked opposite the cartilages of the third rib and along the upper third of the sternum; second sound dull, somewhat prolonged.

The symptoms had commenced eight years previously. During the Summer of 1827, whilst using great muscular exertion, (pumping water,) she was seized with sudden and severe pain at the sternum, attended with violent action of the heart, and a sense of suffocation. The pain increased in violence, and after remaining fixed in front of the chest for two weeks became lancinating, extending from the sternum to the back, and was attended by a short cough. The pain continued for nearly three months, when the dyspnœa increased. This cough, and palpitation continued with varying severity, and attended with occasional œdema of the lower limbs, up to the time of her admission into hospital.

We need not particularise the treatment nor the details of the case. Suffice it that the dyspnœa, &c. increased, and, on the 26th of January, the patient died.

*Dissection.* We pass over other circumstances, to arrive at the state of the heart and the aorta.

" *Heart* much enlarged, more than double its natural size; right cavities more dilated than those of the left; coagula in both ventricles, especially the right. The parietes of the left ventricle measure seven-eighths of an inch in thickness, those of the right ventricle natural. The semilunar valves of the aorta partially ossified; the mitral valves opaque, thickened, with cartilaginous depositions on the free edges; semilunar valves of the pulmonary arteries and tricuspid valves, natural. *The aorta* is apparently much dilated, and, when cut into, presents the remarkable appearance of being a double vessel. The internal vessel is the aorta proper communicating directly with the heart, and is nearly surrounded by another vessel of much larger diameter, which, commencing opposite the great sinus of Valsalva, accompanies the aorta until it divides into the primitive iliacs and terminates in a *cul de sac.* The aorta communicates with the external vessel by a valvular fissure half an inch in length, with rounded edges, which penetrates through the serous and *partly* through the middle coats, and which is situated half an inch above the semilunar valves. The external vessel has no communication with the heart except by this opening. The innominata, subclavian, and left carotid arteries have each double orifices communicating with the aorta and external vessel. The innominata near its mouth is divided by a septum into two portions; the septum terminates in a semilunar edge half an inch above the aorta. In the left carotid the appearance of double vessels is presented for the space of two inches; each has separate openings, one communicating with the aorta; the other with the external vessel. In the left subclavian, on the contrary, there is no double vessel; the orifices opening into the aorta and external vessel being merely formed by a valvular septum at the mouth of the artery. The intercostals of the right side of the thorax communicate with the aorta, whilst those on the left open into the external vessel. The cœliac, superior and inferior mesenterics, renal, and other arteries given off in the abdomen above the bifurcation into the primitive iliacs, communicate with the aorta. The aorta is perforated by numerous foramina, by which, communication is established between it and the external vessel. Anteriorly the external vessel is composed of three coats; an outer, which is cellular; a middle, formed of muscular circular fibres, and an internal, which resembles the serous tissues, but is of variable thickness and presents various colours in different parts of its extent. The cellular coat and the lamina of muscular fibres are continued around the posterior semi-circumference of the aorta, where the muscular circular fibres uniting with the yellow elastic tissue of that artery form in that portion of it its middle coat. The internal membrane of the external vessel, on the contrary, is reflected upon the anterior semi-circumference of the aorta, and the two vessels are there firmly connected by tendinous bands resembling chordæ tendineæ, which pass from one vessel to the other. These bands being cut, the lining membrane may be readily dissected up; it is of a dull white color, semi-transparent, and evidently takes its red and yellow appearance from the subjacent red fibres of the external coat and from the elastic tissue of the aorta. The structure of the aorta in its posterior semi-circumference is normal; in its anterior circumference, the yellow elastic tissue is devoid of the external muscular fibres; the cellular coat is also wanting and is replaced by the reflected membrane of the outer vessel. Numerous ossific deposits exist in the aorta between its serous and elastic coats, but none in the external vessel. Immediately above the bifurcation into the primitive iliacs the external vessel ceases—the red muscular circular fibres and yellow elastic coat become firmly united in the entire circumference of the aorta, and the structure of the iliacs and that of the other arteries throughout the body present the usual arterial formation."

On referring the preparation to Dr. Horner, Professor of Anatomy in the University of Pennsylvania, the latter gentleman communicated it as his opinion, that, at some period, a laceration of the internal and middle coats occurred in the great sinus of Valsalva; and that a column of blood was introduced under the cellular coat so as to detach its semi-circumference from the middle coat down to the primitive iliacs, and also produce a similar condition in the roots of the large branches from the summit of the arch of the aorta.

"Further dissection showed the identity of structure of the middle coat of the external or aneurismal vessel with that of the aorta, and the intimate union of the two in the posterior semi-circumference of the artery. This induced the idea that the blood which had been propelled through the laceration near the sinus of Valsalva had not penetrated the entire thickness of the middle coat of the aorta, but had separated its external from the internal lamina. In order to ascertain whether a separation of this kind could be effected by a fluid thrown between the laminæ of the middle coat, experiments for that purpose were instituted. A small tube with a capillary extremity was introduced between the laminæ of this coat of the artery, and water was forced through it from a syringe in a direction parallel to the sides of the vessel. The result was, that the middle coat was separated in three distinct laminæ.* These views and facts were submitted to the examination of Professor Horner, who fully agreed with me in the idea, that the factitious vessel was the result of the separation of the external from the internal lamina of the middle coat, and that its internal membrane was formed by coagulable lymph, which had simulated the appearance of a serous tissue.

This case may, therefore, be regarded as analogous to those cases of dissecting aneurysms reported by Morgagni, Nicholls, Laennec, Guthrie, M'Lacklin, Shekelton, but differing from them in this, that the aneurism in this instance was formed *between* the laminæ of the middle coat, and that the blood in circulating through the factitious vessel supplied the intercostal arteries of the left side. My search for a parallel case has been unsuccessful, and I am compelled to consider that if this form of aneurism be not *unique*, it must be of extremely rare occurrence. The remarkable lesion of the innominata and left carotid arteries, where the blood after separating the tunics of these vessels as in the aorta, and forming two

* "A specimen in my possession prepared in the manner above mentioned by my friend Dr. Bush of Wilmington, Del., whilst resident physician of the Hospital, shows the separation very satisfactorily."

channels for its passage returns through another rent into the canals of the arteries, is similar to the two cases reported by Mr. Shekelton."

Dr. Pennock remarks that the disease probably had its origin, eight years before the patient's death, during her violent exertions in pumping.

A coloured plate accompanies the description, but the latter is intelligible enough without it.

*Case* 2. "In January, 1836," says Dr. Goddard, "I was requested by Dr. William Harris to make an examination of the body of a woman who had died under the following circumstances: This woman, who was cook in a respectable family in this city, was taken suddenly ill about five o'clock in the afternoon, whilst making some exertion, and complained of faintness and oppression in the region of the heart. Dr. Harris was immediately sent for, and caused her to be bled, which relieved her considerably. He saw her again in the evening and found her weak, but observed no symptoms indicative of immediate danger. He was called up to her, however, in the night, and found her moribund; death took place soon after midnight

On examination, the pericardium was found distended with dark blood, firmly coagulated, estimated to amount to at least eight ounces.

The heart was large and fat, but its structure was normal in every part; the lining membrane of the aorta presented a yellowish appearance, studded here and there with minute ossific patches; about three-fourths of an inch from the semilunar valves a rupture was found nearly an inch in length in a transverse direction, which extended through half the thickness of the middle coat. A channel led both upwards and downwards from this point, which was produced by the separation of the laminæ of the middle coat, extending in width to one-half the circumference of the artery. The upper channel followed the arch of the aorta, and descended as far as the origin of the eighth intercostal artery, leaving the aorta at the summit of the arch to run some inches between the coats of the innominata, left primitive carotid and subclavian. It also ran along some of the intercostals. Many obstacles were thrown in the way of a more perfect dissection by the family, and the distance to which it extended in the vessels of the neck was not precisely ascertained.

The whole of this channel was occupied by a coagulum of dark blood. The lower channel, which appeared to be subsequently formed, and in all probability caused the death of the patient, extended from the rupture in the internal coat to the point of junction of the fibrous pericardium with the root of the aorta; it passed between the two, and then, by a rupture of the serous pericardium, escaped into its cavity."

Dr. Goddard remarks:—

"I believe that if the rupture had not extended into the pericardium, the woman would have lived, and an adventitious serous lining being formed for the new channel, it would have presented, in after-years, the same appearance as Dr. Pennock's preparation. There is one point very remarkable. In Dr. Pennock's case, there are seen in the angle between the new and the old channel, on either side, a number of filaments covered with the new serous lining and extending from the old vessel to the new; in my preparation, the same filaments exist, formed of shreds of the middle coat, but smaller than in Dr. Pennock's, in consequence of the want of the adventitious covering."

